# Targeting the Isoprenoid Biosynthetic Pathway in Multiple Myeloma

**DOI:** 10.3390/ijms24010111

**Published:** 2022-12-21

**Authors:** Staci L. Haney, Sarah A. Holstein

**Affiliations:** Department of Internal Medicine, University of Nebraska Medical Center, 986840 Nebraska Medical Center, Omaha, NE 68198, USA

**Keywords:** multiple myeloma, isoprenoid biosynthetic pathway, prenylation, drug development

## Abstract

Multiple myeloma (MM) is a plasma cell malignancy for which there is currently no cure. While treatment options for MM have expanded over the last two decades, all patients will eventually become resistant to current therapies. Thus, there is an urgent need for novel therapeutic strategies to treat MM. The isoprenoid biosynthetic pathway (IBP) is responsible for the post-translational modification of proteins belonging to the Ras small GTPase superfamily, such as Ras, Rho and Rab family members. Given the important roles these GTPase proteins play in various cellular processes, there is significant interest in the development of inhibitors that disturb their prenylation and consequently their activity in MM cells. Numerous preclinical studies have demonstrated that IBP inhibitors have anti-MM effects, including the induction of apoptosis in MM cells and inhibition of osteoclast activity. Some IBP inhibitors have made their way into the clinic. For instance, nitrogenous bisphosphonates are routinely prescribed for the management MM bone disease. Other IBP inhibitors, including statins and farnesyltransferase inhibitors, have been evaluated in clinical trials for MM, while there is substantial preclinical investigation into geranylgeranyl diphosphate synthase inhibitors. Here we discuss recent advances in the development of IBP inhibitors, assess their mechanism of action and evaluate their potential as anti-MM agents.

## 1. Introduction

Multiple myeloma (MM) is a hematological malignancy classified by the abnormal expansion of clonal plasma cells in the bone marrow and the presence of monoclonal proteins (MP) in the blood and urine. The uncontrolled proliferation of plasma cells and secretion of MP is associated with lytic bone disease, kidney damage, hypercalcemia, anemia and immune suppression. In 2022, it is estimated that 34,470 new cases will be diagnosed and 12,640 deaths will occur from MM [1]. Fortunately for patients, treatment for MM has evolved greatly over the last few decades. The implementation of autologous stem cell transplantation, in conjunction with immunomodulatory agents (IMiDs), proteosome inhibitors and monoclonal antibodies, have greatly lengthened survival. However, MM remains incurable and the overall five-year survival rate is 55% [2]. Despite initial response to treatment, most patients with MM will experience relapse and/or become refractory to current drugs options. The development of novel therapies that target the unique pathophysiology of MM is urgently needed. 

## 2. Overview of the IBP Pathway

**Figure 1:**
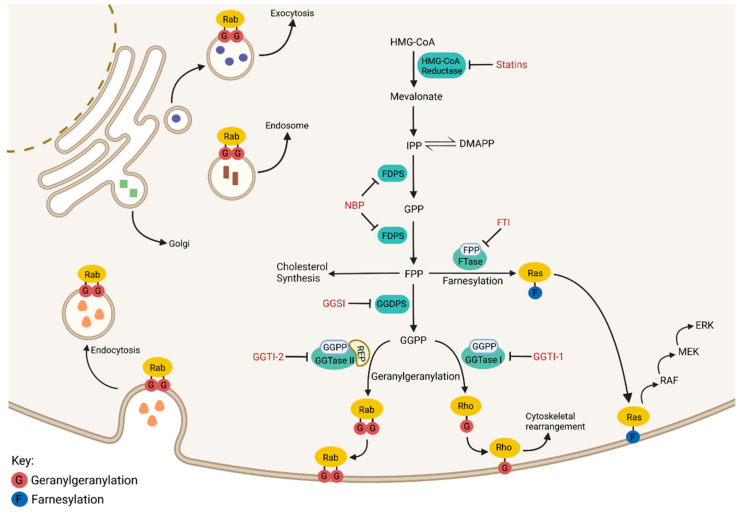
The Isoprenoid Biosynthetic Pathway. An overview of the isoprenoid biosynthetic pathway (IBP) is presented above with inhibitors shown in red and enzymes shown in green. Outcomes of the IBP can be broken down into four categories: (1) cholesterol synthesis, (2) Ras family member farnesylation, (3) Rho family member geranylgeranylation, and (4) Rab family member geranylgeranylation. Ras, Rho and Rab proteins require prenylation to perform their normal cellular functions.

The isoprenoid biosynthetic pathway (IBP), also referred to as the mevalonate pathway, is the most heavily targeted pathway in medicine, with approximately 15 million Americans currently prescribed statins for the treatment of hyperlipidemia. The IBP is responsible for the production of both sterol and non-sterol isoprenoids (Figure 1). The IBP begins when HMG-CoA reductase (HMGR) converts 3-hydroxy-3-methylglutaryl-coenzyme A (HMG-CoA) to mevalonate via the rate-limiting step in the pathway. Mevalonate is sequentially phosphorylated and decarboxylated to produce isopentenyl pyrophosphate (IPP), which can reversibly isomerize to dimethylallyl pyrophosphate (DMAPP). IPP and DMAPP serve as substrates for the enzyme farnesyl diphosphate synthase (FDPS), which generates both the 10-carbon geranyl pyrophosphate (GPP) and the 15-carbon farnesyl pyrophosphate (FPP). FPP is utilized by the enzyme squalene synthase to generate squalene which ultimately leads to the production of sterols. The enzyme geranylgeranyl diphosphate synthase (GGDPS) mediates the condensation of FPP and IPP to produce the 20-carbon geranylgeranyl diphosphate (GGPP). 

FPP and GGPP serve as substrates for farnesyl transferase (FTase) and geranylgeranyl transferases (GGTase I, II, III), respectively. These enzymes facilitate the posttranslational modification (prenylation) of proteins, many of which belong to the Ras small GTPase superfamily of proteins (e.g., Ras, Rab, and Rho families). Prenylation refers to the addition of a 15-carbon isoprenoid chain (via farnesylation) or a 20-carbon isoprenoid chain (via geranylgeranylation) onto a carboxy terminal cysteine residue of a protein. Small GTPases such as the Rho proteins are geranylgeranylated by GGTase I while the family of Rab GTPases are geranylgeranylated by GGTase II [3]. GGTase III is a newly identified prenyltransferase that is responsible for the geranylgeranylation of ubiquitin ligase FBXL2 and Golgi SNARE protein Ykt6 [4,5]. While Ras proteins are typically farnesylated, in the event FTase is inhibited, some Ras proteins (particularly K-Ras) may be geranylgeranylated by GGTase I [6]. This crossover is likely due to the consensus C-terminal sequence (the “CAAX box”), which dictates enzyme specificity and is similar between substrates of FTase and GGTase I. Conversely, GGTase II is unable to recognize its target Rab protein directly and requires the facilitator protein termed REP (Rab escort protein) to deliver substrate to the enzyme’s active site for prenylation [7]. The majority of Rab proteins exhibit a prenylation motif at their C-termini containing two cysteine residues, both of which are prenylated by GGTase II [8]. Conversely, Rab8, Rab13 and Rab23 all contain a CAAX box similar to Rho and Ras proteins and are consequently monoprenylated [9]. Prenylated proteins are dependent on the isoprenoid modification to confer appropriate membrane localization, thus in that manner, the activity of Ras small GTPase proteins is regulated via their prenylation [10,11]. 

The Ras small GTPase superfamily includes over 150 proteins that play a myriad of significant functions within the cell. Rab proteins control virtually all aspects of membrane trafficking within the cell, including facilitating vesicle budding, motility, docking, and fusion (Figure 1). Mutated Rab proteins that cannot be geranylgeranylated are mis-localized and non-functional, indicating that prenylation regulates Rab activity, and by extension, protein trafficking [10]. Inhibition of Rab geranylgeranylation induces the cytosolic accumulation of unmodified Rab proteins [12]. The Rho-family of GTPases integrate signals from the cells’ physical environment to mediate the reorganization of the actin cytoskeleton, allowing for the reshaping and migration of cells [13]. Non-prenylated Rho proteins are inactive and sequestered to the cytosol via the RHO-specific guanine nucleotide dissociation inhibitors [14]. Lastly, Ras family proteins serve as activation nodes to various signaling networks that regulate cell proliferation, gene expression, cell survival and differentiation [15]. For instance, Ras prenylation and subsequent membrane localization initiates activation of the MAPK signaling pathway, which is heavily tied to tumor cell pathogenesis (Figure 1). Due to the diverse role of GTPase proteins in cellular processes, particularly in malignant cells, there has been considerable interest in the development of inhibitors that disrupt their prenylation and thus their activity in cancer cells.

Several lines of investigation demonstrate that inhibitors of the IBP have pleiotropic effects in MM cells and may serve as a novel therapeutic approach. This review will carefully evaluate the potential of inhibitors at each step in the IBP and summarize where the field currently stands regarding the development of IBP inhibitors for MM therapy.

## 3. Classes of IBP Inhibitors

**Figure 2:**
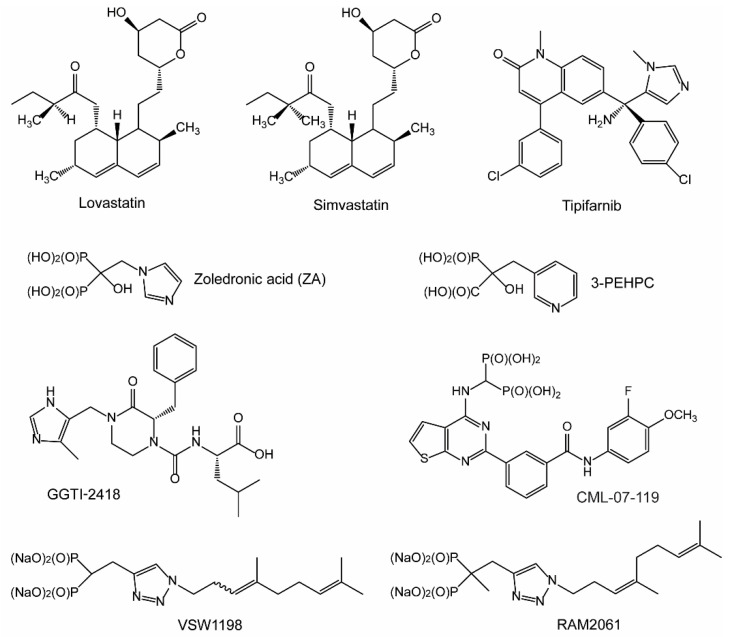
Isoprenoid biosynthetic pathway (IBP) and prenyltransferase inhibitors. The chemical structures of select inhibitors are shown above.

**Statins:** As mentioned previously, statins are widely prescribed for the management of high cholesterol. Statins inhibit HMGR, which performs the rate limiting step and governs entry into the IBP. While statins inhibit endogenous cholesterol synthesis, their cholesterol lowering effects are largely the result of upregulation of the LDL-receptor and subsequent decrease in circulating LDL levels [16,17]. In addition, statins may influence cardiovascular health via cholesterol-independent mechanisms, such as reduction in vascular inflammation, increased endothelial function, and stabilization of atherosclerotic plaques [18]. Inhibition of isoprenoid synthesis and subsequent disruption in protein prenylation is believed to be responsible for the cholesterol-independent effects of statins [18]. Statins deplete the downstream production of the isoprenoids FPP and GGPP, and for this reason are widely used in vitro to study the role of prenylation in the modulation of Ras small GTPase proteins, as they disrupt both farnesylation and geranylgeranylation [19]. Numerous in vitro studies have illustrated that statins induce apoptosis in MM cell lines [20,21,22,23]. These findings have led many to theorize that statin treatment could be utilized to make an immediate impact in the care of patients with MM. To date, both lovastatin and simvastatin (Figure 2) have been evaluated in clinical trials for MM [24,25,26].

**NBPs:** After HMGR, the next druggable target in the IBP is FDPS. Bisphosphonates (BP), and the later characterized nitrogenous bisphosphonates (NBPs), are potent inhibitors of the enzyme FDPS. Structurally, BPs are non-hydrolysable analogues of naturally occurring pyrophosphates found in the bone matrix. NBPs display a strong affinity for bone and have minimal systemic exposure, making them potent inhibitors of bone resorption and bone remodeling activity, with limited potential for off target side-effects [27,28]. NBPs disrupt formation of the osteoclast ruffled border via their effects on cytoskeletal rearrangement and disruption of the F-actin ring [29,30,31]. Early BPs were first evaluated in clinical trials for the treatment of MM bone disease in 1980, a decade before their molecular mechanism or target were even identified [32]. Since this time, NBPs have become a mainstay in the treatment of MM bone disease, as they inhibit bone destruction and reduce skeletal-related events in patients with MM [33,34]. Zoledronic acid (ZA) is a NBP and FDPS inhibitor that is routinely prescribed for the management of MM bone disease (Figure 2). 

**FTase and Ras inhibitors:** FTase-mediated farnesylation is the first irreversible, rate-limiting step for Ras membrane association [35]. The Ras family of proteins, including H-, K-, and N-Ras, play essential roles in regulating cellular proliferation and survival in both normal and cancer cells. Membrane bound Ras triggers RAF-kinases, which leads to subsequent phosphorylation and activation of MEK and ERK-kinases. Upregulation of the RAS/RAF/MEK/ERK pathway (also referred to as the MAPK pathway) is a prominent molecular feature of MM and is driven by myeloma-induced changes in the bone that result in elevated levels of interleukins [36]. Furthermore, Ras mutations are consistently observed across a range of cancer types, including MM where approximately 23% of patient samples contain Ras mutations [37]. In particular, K-Ras mutations are associated with poor prognosis in MM [38]. Due to its importance in disease pathogenesis, significant interest has been placed on the development of Ras inhibitors as anti-cancer agents. Unfortunately, development of Ras inhibitors has proven problematic, and to date only one specific inhibitor of G12C mutated K-Ras (a mutation rarely observed in MM) has been approved [39]. Many have theorized that FTase inhibitor (FTI)-mediated disruption in Ras prenylation may serve as an alternative approach by which to modulate oncogenic Ras activity. To date, four FTIs have been evaluated in clinical trials and have shown activity in breast cancer, myelodysplastic syndrome, and leukemia [40,41,42,43]. Tipifarnib (also referred to as R115777) is an imidazole-containing heterocyclic compound and the first FTI to be evaluated in clinical trials for MM [44,45] (Figure 2). 

**GGTase inhibitors:** As mentioned previously, the enzyme GGDPS produces the isoprenoid GGPP which is utilized in the prenylation reaction of Rho and Rab proteins by GGTase I and GGTase II, respectively. Prenylation of Rho GTPases promotes their membrane localization and GTP loading, leading to activation of signaling pathways that direct actin polymerization [46] (Figure 1). Rho proteins play a role in various aspects of pathogenesis, including cancer cell migration and invasion [47]. In fact, inhibition of Rho-C suppresses proliferation of RPMI-8226 MM cell lines in vitro [48]. One hurdle for the development of GGTase I inhibitors (GGTI-1) is the ability to eliminate overlap with FTase, as both enzymes share considerably similarity within their active site. Only one GGTI-1 (GGTI-2418) has entered phase I clinical trials, albeit not for MM [49] (Figure 2). Other GGTI-1 compounds have been evaluated in vitro, but overall, the investigation into their cellular effects in MM cells is incomplete. 

GGTase II is responsible for the geranylgeranylation of Rab proteins, which act as mediators of protein trafficking in the cell. The development of potent GGTase II inhibitors (GGTI-2) worthy of translation to clinical trials has been difficult to achieve. This is largely due to the unique way that GGTase II only recognizes dimers of Rab and REP during the prenylation reaction. Still, several GGTI-2 compounds have been developed and evaluated in preclinical studies. 3-PEHPC (Figure 2) was the first GGTI-2 to be characterized and is an analogue of the potent BP known as risedronate [50,51,52]. In MM cell lines, 3-PEHPC induces concentration-dependent induction of apoptosis and prevents geranylgeranylation of Rab6 [51]. However, its lack of potency (IC_50_ against GGTase II of 600 μM) largely limit its use to in vitro studies [50]. Additional structure–function analysis of 3-PEHPC analogs found that substitution at the 6th position of the heterocyclic ring yields GGTI-2 compounds with enhanced potency [53]. In an effort to optimize drug-like properties, additional GGTI-2 compounds were synthesized, including triazole based inhibitors, N-oxide derivatives of 3-PEHPC and benzimidazole carboxyphosphonates. However, all showed biological activity in enzyme assays in the micromolar range [54,55,56]. Using tetrahydrobenzodiazepine as a scaffold, researchers were able to convert a dual FTI/GGTI-2 into a potent and selective GGTI-2 compounds with nanomolar activity (IC_50_ = 49 nM) [57]. While this compound inhibited the proliferation of various cancer cell lines in vitro, its effects on MM cells has not been evaluated. Despite the nanamolar potency, the aforementioned GGTI-2 compound has not advanced to clinical trials for unknown reasons. Of note, inhibitors for specific Rab proteins, such as Rab8a, have been developed and may serve as an alternative mechanism by which to target Rab activity [58].

**GGDPS inhibitors:** GGDPS inhibitors (GGSI) deplete cellular pools of GGPP and serve as an alternative approach to target downstream geranylgeranylation of both Rho and Rab proteins. Several GGSI compounds have been developed in recent years, with some of the most potent being isoprenoid triazole bisphosphonate compounds. The GGSI VSW1198 inhibits geranylgeranylation at concentrations as low as 30 nM in MM cell culture and is highly specific for GGDPS over other enzymes in the IBP pathway [59] (Figure 2). VSW1198 is a 3:1 mixture of homogeranyl and homoneryl isomers that interact in a synergistic manner to inhibit GGDPS [60,61]. Additional structure–function studies utilizing triazole bisphosphonate compounds led to development of the α-methylated derivatives RAM2093 (homogeranyl isomer) and RAM2061 (homoneryl isomer, Figure 2). Both isomers display similar IC_50_ values in enzyme assays (RAM2061 = 86 nM and RAM2093 = 125 nM) and have a nanomolar activity in MM cells [62]. Evaluation of C-2-substituted thienopyrimidine-based bisphosphonates identified the GGSI CML-07-119 which blocks the proliferation of RPMI-8226 cells (EC_50_ = 90 nM) and has a strong affinity for GGDPS over FDPS [63]. While a GGSI has not yet advanced to clinical trials, the recent development and preclinical characterization these new compounds give hope to their eventual translation to the clinic. 

## 4. Cellular Effects of IBP Inhibitors

The cellular effects of IBP inhibitors in MM cells have been well documented in the literature, with most reports citing a prenylation-dependent mechanism. Statins are widely used to study the role of prenylation as they deplete cellular levels of FPP and GGPP causing disruption of both farnesylation and geranylgeranylation. Multiple in vitro studies have linked the depletion of GGPP to the anti-MM effects of statins. For instance, in statin-treated MM cell lines, the addition of GGPP, but not FPP, rescues cells from statin-induced apoptosis [23,64,65,66]. In one of these studies, statins were shown to disrupt light chain trafficking in MM cells, leading to accumulation of light chain in the ER, activation of the unfolded protein response pathway (UPR) and induction of caspase-mediated apoptosis [23]. The UPR is a cell survival mechanism activated by accumulation of proteins within the ER. When initiated, the UPR triggers several protective measures to aid in the clearance of ER proteins, including reduction in protein synthesis and upregulation of ER chaperones and folding enzymes [67]. In the event ER stress is sustained, the UPR may activate apoptosis. Due to their continuous production and secretion of MP, MM cells express near maximal levels of proteins associated with the UPR and thus have a lower threshold for induction of the pro-apoptotic arm of the UPR [68]. 

**Figure 3:**
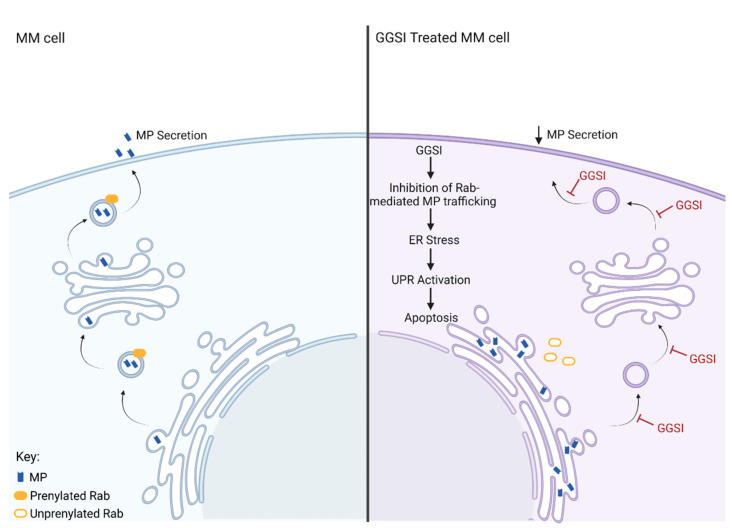
Cellular effects of GGSI in an MM cell. The cell panel on the left depicts normal Rab-mediated secretion of monoclonal protein (MP) from an MM cell. The panel on the right shows the effects of GGSI on MP secretion. In the GGSI-treated MM cell, Rab-mediated MP trafficking is inhibited, leading to ER stress caused by accumulation of MP within the ER, activation of the UPR, and ultimately apoptosis.

Similar to statin treatment, inhibition of GGTase II, but not FTase or GGTase I, induces accumulation of intracellular light chain and UPR mediated apoptosis in MM cell lines [23]. Given that Rab proteins are the only known substrate of GGTase II, this would suggest that disruption of Rab geranylgeranylation is responsible for the accumulation of light chain induced by statin treatment. Inhibition of Rab geranylgeranylation can be achieved by direct disruption of GGTase II activity or by upstream inhibition of GGDPS via a GGSI and depletion of GGPP. In MM cell lines, treatment with the GGSI RAM2061 induces upregulation in UPR markers (ATF4, PERK, IRE1 and CHOP) and induction in caspase-mediated apoptosis [69]. Importantly, GGSI-mediated activation of the UPR pathway in MM cells is recapitulated by a GGTI-2, indicating disruption of Rab geranylgeranylation is the mechanism responsible for the observed cytotoxic effects of GGSI treatment [23]. In addition, studies in other malignancies such as pancreatic adenocarcinoma, Ewing sarcoma and osteosarcoma have revealed that treatment with a GGTI-2, but not with a GGTI-1, recapitulates the effects of RAM2061 in inducing the UPR and apoptosis [70,71]. In addition, co-incubation with GGPP fully prevents the anti-proliferative effects of GGSI treatment in MM cells [72]. Inhibition of Rab-mediated MP trafficking and subsequent activation of the pro-apoptotic arm of the UPR is central to the anti-MM activity of GGSIs (Figure 3). 

Depletion of GGPP is also linked to the cytotoxicity of FDPS inhibitors, such as ZA. In MM cell lines, ZA-induced apoptosis is reversed by the addition of GGPP [73]. Furthermore, reports suggest that NBP-mediated osteoclast dysfunction is the result of GGPP depletion and occurs independent of FTase activity [31]. Coxon et al. demonstrated that treatment with a GGTI-1 (but not an FTI) prevents osteoclast formation, disrupts osteoclast cytoskeletal rearrangement, induces osteoclast apoptosis, and inhibits bone resorption [31]. Thus, GGDPS may serve as a more targeted approach for the treatment of both primary disease (through inhibition of Rab-mediated MM trafficking) and MM-mediated bone destruction (through inhibition of osteoclast activity). While GGSIs have not been evaluated for their potential effects on bone, GGTase II inhibition has been shown to inhibit bone resorption in vitro [50,74].

Farnesylation-dependent effects cannot be ruled out as a putative mechanism, as several studies have reported changes in Ras activity following IBP inhibitor treatment. One report found that incubation with either fluvastatin or simvastatin downregulated signal transduction of Ras/ERK and Ras/Akt pathways in mouse MOPC-31C MM cells. This resulted in perturbed expression and secretion of MIP-1α, an important regulator in the development of MM osteolytic bone disease [75]. Another study demonstrated that while FTI efficacy was limited due to alternative prenylation of K-Ras and N-Ras, combination treatment of either FTI or GGTI-1 with lovastatin synergistically induced apoptosis and inhibited MM cell proliferation, migration, K- and N-Ras processing and RAS-to-MAPK signaling [76]. Furthermore, co-treatment of RPMI-8226 MM cells with an FTI and GGTI-1 caused enhanced cell death and greater inhibition in K-Ras processing [77]. Such findings suggest that combining FTI with GGTI-1 or statin therapies may inhibit the alternative prenylation of oncogenic Ras proteins and provide synergistic anti-MM effects.

Limited evidence exists to suggest that IBP inhibitors induce prenylation-independent effects. In one study, the results of western blot analysis showed that simvastatin-induced S-phase cell cycle arrest was associated with activation of the Chk1–Cdc25A–cyclin A/CDk2 pathway and that silencing Chk1 expression inhibited simvastatin-mediated effects in MM cell lines [78]. Collectively, the literature would indicate a prenylation-dependent mechanism for IBP inhibitors, but further research into their effects in cancer cells is warranted. 

## 5. Combination Studies

Current treatment protocols for MM include multi-drug combination therapies, thus understanding how IBP inhibitors interact with available therapeutic agents is critical. IMiDs, such as thalidomide, lenalidomide and pomalidomide, are routinely prescribed for the treatment of MM. Co-incubation of MM cell lines with simvastatin and lenalidomide resulted in synergistic reduction in cell viability and induction in caspase-8 cleavage and down regulation in pStat3 [79]. GGPP and mevalonate add-back experiments illustrate a prenylation-dependent mechanism underlying these effects. In another in vitro study, thalidomide was shown to enhance the pro-apoptotic effects of simvastatin and lovastatin [80]. Combination thalidomide and statin treatment also reduced cell migration and inhibited VEGF and MMP-9 expression in MM cells. Similarly, the cytotoxic effects of several IBP inhibitors (lovastatin, ZA or GGSI) were enhanced by the addition of thalidomide in MM cell lines [66]. These data provide a rationale for the clinical evaluation of IMiDs and IBP inhibitors in patients with myeloma. 

Bortezomib is a first-in-class proteosome inhibitor and has been a breakthrough for the treatment of MM. Notably, in vitro synergy between bortezomib and multiple classes of IBP inhibitors has been documented in the literature. One study demonstrated that the FTI tipifarnib has synergistic anti-MM effects when used in combination with bortezomib [81]. The authors demonstrated that FTI/bortezomib treatment enhanced the activation of the ER stress response genes and overcame cell adhesion–mediated drug resistance [81]. In another study, synergy with bortezomib was linked to tipifarnib’s ability to inhibit the degradation of bortezomib-induced aggresomes, resulting in further protein accumulation and enhanced apoptosis in MM cells [82]. In addition, fluvastatin treatment synergized with bortezomib to enhance apoptosis and upregulation of stress response genes (namely ATF4, ATF3, and CHOP) in t(4;14)-positive MM cells [83]. Lastly, combination GGSI and proteosome inhibitor (bortezomib or carfilzomib) treatment potentiates activation of the UPR and apoptotic pathways, as well as induces upregulation of markers associated with the immunogenic cell death pathway in MM cell lines [84]. Based on these findings, further clinical assessment of proteosome inhibitors with IBP inhibitors is clearly warranted.

## 6. In Vivo Studies

An array of studies utilizing mouse models of MM have demonstrated the in vivo efficacy of IBP inhibitors (Table 1). In a tail vein model using KMS11 luciferase-expressing MM cells, atorvastatin treatment was able to significantly reduce tumor burden and lengthen survival in NOD/SCID mice [85]. In a plasmacytoma model using human INA-6 MM cells, mice treated with the FDPS inhibitor ZA displayed prolonged survival relative to control animals [86]. Furthermore, induction of apoptotic markers and blockage of prenylation was visible in tumor tissue isolated from ZA treated mice [86]. In another study, 3-PEHPC prevented the development of osteolytic bone lesions and reduced tumor burden in the 5T2MM mouse model of MM [52]. A recently developed GGSI (CML-07-119) was shown to reduce serum M-protein and disrupt Rap1a prenylation following a 14-day treatment period in Vk*MYC mice [87]. Lastly, another GGSI (RAM2061) was shown to slow tumor growth in a flank xenograft model using MM.1S cells, as well as disrupt geranylgeranylation in vivo [69]. 

While the before-mentioned studies demonstrate that IBP inhibitor treatment alone impedes tumor growth, combination treatments have also been shown to display potent anti-MM effects in vivo. Dual inhibition of the IBP through use of a GGSI (VSW1198) and lovastatin significantly slowed tumor growth in the MM.1S flank xenograft model and reduced hepatic GGPP levels to an undetectable level [88]. In the same xenograft model, combination GGSI treatment (RAM2061) and bortezomib synergistically slowed tumor growth and reduced plasma MP levels in mice relative to single agent controls [84]. In addition, co-treatment with fluvastatin and bortezomib significantly slowed tumor growth in NOD/SCID mice injected with t(4;14)-positive NCI-H929 cells [83]. Lastly, researchers demonstrate that co-administration of an FTI (R115777) and a Chk1 inhibitor (UCN-01) reduced tumor burden and induced apoptosis in the MM.1S flank xenograft model [89]. These finding warrant further preclinical evaluation of IBP inhibitors in combination with other agents for the treatment MM.

## 7. Clinical Trials and Epidemiological Studies

Despite their decades of use, clinical trials evaluating the use of statins in MM populations are limited and results are mixed (Table 2). In the first phase II trial, six patients with MM that were refractory to two cycles of bortezomib or bendamustine were concomitantly administered simvastatin (80 mg/daily) during an additional two cycles of chemotherapy (NCT00399867). Five out of six patients who received both simvastatin and bortezomib/bendamustine saw reduction in plasma MP levels, suggesting that simvastatin might reduce drug resistance in MM [24]. Conversely, a phase II trial utilizing simvastatin (15 mg/kg/day for 7 days followed by a rest period of 21 days in two 4-week cycles) in six heavily pretreated patients with MM failed to show reduction in plasma MP levels [25]. In addition, markers of osteoclast activity (tartrate resistant acid phosphatase, TRACP) and bone resorption (NTX collagen fragments) transiently increased in all patients during the administration of simvastatin, suggesting that this treatment method may be detrimental to patients with MM. It is important to note that both before-mentioned studies were limited in their scope and not sufficient to conclude or refute efficacy of simvastatin. In a larger study, heavily pre-treated patients with MM were administered thalidomide and dexamethasone (TD) with or without lovastatin (L) over a period of six 28-day cycles (2 mg/kg on days 1–5 and 8–12 and 0.5 mg/kg on days 15–28) [26]. A clinical response (defined as at least 50% reduction in serum MP) was observed in 32% of TD patients and 44% of TDL patients (TD *n* = 42, TDL *n* = 49). Furthermore, lovastatin use was associated with a decrease in the median time to response (TDL = 1.5 month, TD = 3 months, *p* = 0.001) and longer progression free survival (TD = 16 months, TDL = 33 months, *p*  =  0.04849). 

The failure of statins in some clinical trials may be the result of sub-therapeutic doses that are insufficient to disrupt prenylation in vivo, as standard doses of statins produce sub-micromolar concentrations in the plasma [90]. As a whole, studies that evaluate statin activity in cancer cell lines primarily utilize drug concentrations in the micromolar range [91]. Two phase I studies have evaluated the safety of high dose lovastatin in patients with advanced malignancies other than MM [92,93]. In the first study, lovastatin was administered to cancer patients at a range of 2 mg/kg/day to 45 mg/kg/day during a 7-day course given monthly [92]. While serum micromolar levels of lovastatin were observed in some patients (range 0.1–3.9 μM), the treatment strategy was associated with dose-limiting toxicities. In another phase I trial, patients received a 4-day course of lovastatin given monthly (range of 10–415 mg/m^2^/dose) [93]. Importantly, dose-limiting toxicity was not observed, and lovastatin levels in the plasma ranged from 0.06 μM to 12.3 μM. While these studies illustrate that administration of high dose lovastatin is possible, concern remains about off-target toxicities. 

**Table 2:** Summary of clinical trials for IBP inhibitors.

Type	Study Design	Treatment	Outcomes	Cit.
Phase II	MM patients refactory to bortezomib (bort) or bendamustin (ben) were adminstered simvastatin + bort/ben (*n* = 6)	Simvastatin (80 mg/daily) for two cycles + bort/ben	Reduction in plasma MP levels in 83% (5/6) patients	[24]
Phase II	Heavily treated MM patients administered simvastatin (*n* = 6)	Simvastatin (15 mg/kg/day for 7 days + 21 day rest period in two 4-week cycles)	No change in plasma MP levels and a transient increase in markers of bone resorption	[25]
Phase II	Heavily pretreated MM patients administered thalidomide and dexamethasone (TD) with or without lovastatin (L). (TD *n* = 42, TDL *n* = 49)	Lovastatin (28 day cycles of 2 mg/kg on days 1–5 and 8–12, 0.5 mg/kg on days 15–28)	Significant reduction in serum MP observed in 32% of TD and 44% of TDL patients. Improvement in medium time to repsonse and longer progression free survival in TDL group.	[26]
Phase I	Patients with solid tumors treated with high dose lovastatin (*n* = 88)	Lovastatin (2 mg/kg/day to 45 mg/kg/day for 7 days given monthly)	Dose limiting toxicity occured. Yielded micromolar plasma concentrations of lovastatin.	[92]
Phase I	Patients with advanced malignancies treated with a range of high dose lovastatin (*n* = 32)	Lovastatin (10 to 415 mg/m2/dose for 4 days given monthly)	No dose limiting toxicity observed. Yielded micromolar plasma concentration of lovastatin. No anti-tumor response.	[93]
Phase III	Newly diagnosed MM patients administered zoledronic acid (ZA, *n* = 981) or clodronic acid (CLO, *n* = 979) with or without chemotherapy	ZA (4 mg every 4 weeks) CLO (1600 mg daily)	Relative to CLO, ZA reduced mortality by 16% and lengthened overall survival by 5.5 months	[33,34]
Phase II	MM patients were treated with tipifarnib (*n* = 43)	Tipifarnib (300 mg twice daily for 3 weeks, every 4 weeks)	Disease stabilization in 64% of patients	[45]

Several retrospective population-based studies concluded that statin use is associated with increased survival in patients with MM, as well as overall lower risk of MM development [94,95,96,97]. To better understand the association between statins and MM risk, a large case-control study utilizing patient data (*n* = 2532) from six health care systems was conducted [98]. This study concluded that statin use (defined as ≥6 months treatment period) 48 to 72 months prior to diagnosis was associated with a reduced risk of MM development, compared to non-statin users (risk ratio of 0.72–0.8). Conversely, recent initiation of statins (<36 months) was not associated with reduction in MM risk (risk ratio range of 0.9–0.99). Furthermore, older patients displayed a stronger association between statin use and reduction in MM risk for all latency periods (risk ratio of 0.67–0.87). Such findings suggest that association between statin treatment and MM risk varies by both exposure window and patient age and further investigation is needed to understand this connection. 

BPs and NBPs have been prescribed for the management of MM bone disease for decades. In a large multi-center clinical trial, newly diagnosed MM patients were administered the NBP zoledronic acid (ZA; 4 mg IV every 4 weeks; *n* = 981) or the BP clodronic acid (CLO; *n* = 979) with or without intensive chemotherapy (Table 2) [33,34]. They found that relative to CLO, ZA reduced mortality by 16% (*p* = 0.0118) and lengthened overall survival by 5.5 months (*p* = 0.04). They concluded that immediate treatment with ZA has therapeutic benefit beyond bone health and improves overall survival independent of its effects on skeletal-related events. Such benefits may perhaps be explained by the fact that NBPs display anti-cancer properties in various cell types, including inhibition of tumor cell proliferation, anti-angiogenesis, disruption in tumor cell invasion, and activation of apoptosis [99,100,101,102]. In addition, ZA activates the effector function of γδ T-cells in patients with MM, indicating it has immunomodulatory effects [103]. Importantly, the ability of ZA to expand γδ T-cells in vitro is fully abrogated by the addition of mevalonate, suggesting such effects are reliant on disruption of the IBP pathway. 

Given the prevalence of K-Ras mutations in MM, targeting Ras farnesylation via FTI treatment has been of considerable interest to researchers. Tipifarnib is one of two FTIs that have advanced to clinical trials and is the first to be evaluated for MM treatment [45]. In a phase II clinical trial, 300 mg tipifarnib was administered to 43 patients with MM with relapsed/refractory disease (Table 2) (NCT00012350). In this study, 64% of patients achieved disease stabilization. Interestingly, while both suppression of FTase (but not GGTase I) and disruption of farnesylation were observed in patient samples, disease stabilization did not correlate with farnesylation status. Such findings are in agreement with in vitro studies showing tipifarnib induces apoptosis in MM cells without disruption in Ras farnesylation [104]. Taken together, these findings suggest alternative pathways outside of inhibition of farnesylation may be responsible for tipifarnib’s activity in MM cells. 

The failure of FTIs in some clinical trials is likely the result of alternative prenylation of oncogenic K-Ras and N-Ras by GGTase I. One possible solution to this problem might be dual inhibition of FTase and GGTase I. A dual specificity inhibitor (DSI) of FTase and GGTase I, termed L-778123, has been developed and evaluated in phase I clinical trials. The DSI successfully disrupted the prenylation of Rap1a and HDJ2 in vivo; however, it failed to block prenylation of K-Ras proteins isolated from patient peripheral blood mononuclear cells, signifying that the intended target of the drug was not inhibited [105]. A series of novel diaryl ether lactams that function as a potent DSI of FTase and GGTase I was later developed, but unfortunately resulted in rapid lethality when tested in animal models [106]. 

## 8. Conclusions

Multiple myeloma remains an incurable disease and novel treatment options are urgently needed. Here we have summarized the considerable body of research that indicates that targeting the IBP pathway may serve as a novel approach for MM therapy. Of note, statin treatment possesses the ability to make an immediate impact in patient care. Clinical trials with statins reveal conflicting results, but overall benefits were observed in select studies. Clinical trials utilizing high-dose statins are needed to fully evaluate the potential of these agents as MM therapeutics. 

The considerable body of research regarding the cellular effects of IBP inhibitors in MM cells would suggest a prenylation-dependent mechanism. In particular, the depletion of GGPP has been linked to the activity of statin, NBP, and GGSI compounds. Statins, GGSI, and GGTI-2 agents have all been shown to disrupt MP trafficking, leading to activation of the UPR and apoptosis in MM cells. This mechanism is likely the result of disruption in Rab geranylgeranylation and subsequent inhibition of Rab-mediated MP trafficking. Currently no therapeutic strategies exist to target MP trafficking in MM and thus disruption of Rab geranylgeranylation serves as a novel therapeutic strategy. GGTI-2 and GGSI therapy offer a more targeted approach to disrupt Rab activity compared to statins. Relative to GGTI-2 compounds, GGSIs have undergone more thorough preclinical evaluation and several mouse models have demonstrated the in vivo anti-MM effects of GGSI treatment. GGSI therapy shows considerable promise, and the recent development and preclinical evaluation of several highly potent GGSI compounds gives reason to believe that clinical translation of these novel anti-MM agents is on the horizon. 

## Figures and Tables

**Table 1:** Summary of in vivo studies utilizing IBP inhibitors.

Model	Mouse Strain	Treatment Groups	Outcomes	Cit.
IV injection of KMS11-Luc cells	NOD/SCID	Solvent control Atorvastatin (10 mg/kg, 3x/wk) Atovastatin (50 mg/kg, 3x/wk)	Decreased tumor burden and prolonged survival in both atorvastatin treated groups	[85]
IP injection of INA-6.Tu1 cells	CB-17 /lcrCRL-scid	Solvent control Zoledronic acid (ZA; 0.08 mg/kg, 3x/wk) Zoledronic acid (ZA; 0.32 mg/kg, 3x/wk)	Prolonged survival in both ZA treated groups	[86]
IV injection of 5T2MM cells isolated from diseased mouse bone marrow	C57BL/KaLwRijHsd	naïve 5T2MM + solvent 5T2MM + risedronate (125 μg/kg 2x/wk) 5T2MM + 3-PEHPC (125 μg/kg 2x/wk)	Reduced osteoclast number, reduced osteolytic lesions, and decreased tumor burden in 3-PEHPC and risedronate treated groups	[52]
VK*MYC transgenic	Aged mice w/measurable disease	Solvent control GGSI (3 mg/kg/day)	Reduction in M protein levels in GGSI treated mice	[87]
Flank injection of MM.1S cells	NOD/SCID	Solvent control RAM2061 (0.08 mg/kg, 2x/wk)	Slowed tumor growth in RAM2061-treated mice	[69]
Flank injection of MM.1S cells	NOD/SCID	Solvent control VSW1198 (0.05 mg/kg 1x/wk) Lovastatin (10 mg/kg daily) VSW1198 + lovastatin	Slowed tumor growth in combination group	[88]
Flank injection of MM.1S cells	NOD/SCID	Solvent control RAM2061 (0.08 mg/kg, 2x/wk) Bortezomib (0.3 mg/kg, 2x/wk) RAM2061 + bortezomib	Slowed tumor growth, prolonged survival and reduction in MP levels in combination group	[84]
Flank injection of NCI-H929 cells	NOD/SCID	Bortezomib (1 mg/kg 2x/wk) Bortezomib + fluvastatin (50 mg/kg)	Slowed tumor growth in combination group	[83]
Flank injection of MM.1S cells	Athymic NCr-nu/nu	Solvent control FTI (25 mg/kg daily) UNC-01 (0.5 mg/kg daily) FTI + UNC-01	Reduced tumor burder in combination group	[89]

## Data Availability

Not applicable.

## Abbreviations

MM (multiple myeloma), IBP (isoprenoid biosynthetic pathway), MP (monoclonal protein), IMiDs (immunomodulatory drugs), HMGR (HMG-CoA reductase), HMG-CoA (3-hydroxy-3-methylglutaryl-coenzyme A), IPP (isopentyl pyrophosphate), DMAPP (dimethylallyl pyrophosphate), FDPS (farnesyl diphosphate synthase), GPP (geranyl pyrophosphate), FPP (farnesyl pyrophosphate), GGDPS (geranylgeranyl diphosphate synthase), GGPP (geranylgeranyl diphosphate), FTase (farnesyltransferase), GGTase (geranylgeranyl transferase), REP (Rab escort protein), BP (bisphosphonate), NBP (nitrogenous bisphosphonate), FTI (FTase inhibitor), GGTI-1 (GGTase-I inhibitor), GGTI-2 (GGTase-II inhibitor), UPR (unfolded protein response), ZA (zoledronic acid), DSI (dual specificity inhibitor).
